# *Gaultheria*: Phytochemical and Pharmacological Characteristics

**DOI:** 10.3390/molecules181012071

**Published:** 2013-09-30

**Authors:** Wei-Rui Liu, Wen-Lin Qiao, Zi-Zhen Liu, Xiao-Hong Wang, Rui Jiang, Shu-Yi Li, Ren-Bing Shi, Gai-Mei She

**Affiliations:** School of Chinese Pharmacy, Beijing University of Chinese Medicine, Beijing 100102, China; E-Mails: liuweirui2012@126.com (W.-R.L.); qiaowenlin07@126.com (W.-L.Q.); lzz332@126.com (Z.-Z.L.); bucm_pharm@126.com (X.-H.W.); jiangrui54264@126.com (R.J.); lishuyi1993@126.com (S.L.); shirb@126.com (R.-B.S.)

**Keywords:** *Gaultheria*, chemical components, pharmacological activity, methyl salicylate glycosides, anti-rheumatic effects

## Abstract

The genus *Gaultheria*, comprised of approximately 134 species, is mostly used in ethnic drugs to cure rheumatism and relieve pain. Phytochemical investigations of the genus *Gaultheria* have revealed the presence of methyl salicylate derivatives, C_6_-C_3_ constituents, organic acids, terpenoids, steroids, and other compounds. Methyl salicylate glycoside is considered as a characteristic ingredient in this genus, whose anti-rheumatic effects may have a new mechanism of action. In this review, comprehensive information on the phytochemistry, volatile components and the pharmacology of the genus *Gaultheria* is provided to explore its potential and advance research.

## 1. Introduction

The genus *Gaultheria* (Ericaceae) is widely distributed around the Pacific Ocean, westwards to western slopes of the Himalayas and the southern areas of India [1]. Most *Gaultheria* species growing in Southwest China are regarded as traditional herbal medicines. Parts of plants in this genus are used by nine minority nationalities for the treatment of wind-damp, as well as relieving pain. Additionally, *G*. *procumbens* is used as a folk remedy in America and Canada, and *G*. *fragrantissima* is employed in India [2]. Modern research has demonstrated that these plants exhibit anti-inﬂammatory, anti-oxidative, antibacterial and analgesic activities. To the best of our knowledge, about 109 compounds were reported from this genus, including methyl salicylate derivatives, C_6_-C_3_ constituents, terpenoids, and steroids. In particular methyl salicylate (**1**) and three methyl salicylate glycosides, methyl salicylate 2-*O*-*β*-d-glucopyranoside (**2**), gaultherin (**3**) and methyl benzoate-2-*O*-*β*-d-xylopyranosyl(1-2)[*O*-*β*-d-xylopyranosyl(1-6)]-*O*-*β*-d-glucopyranoside (**5**), from the aerial parts of *G*. *yunnanensis*, display notable analgesic and anti-inﬂammatory activities, which has impelled a number of studies on the phytochemistry and biology of this genus [3]. Meanwhile, it’s worth mentioning that methyl salicylate glycoside when used in animal experiments to explore its pharmacological effects could overcome the side-effects caused by aspirin in the clinic [3,4,5]. Herein, we summarize the phytochemical and biological studies on the genus *Gaultheria*. What’s more, all the compounds reported in the essential oils of *Gaultheria* are listed below, and the corresponding plants are included as well.

## 2. Chemical Constituents

**Figure 1:**
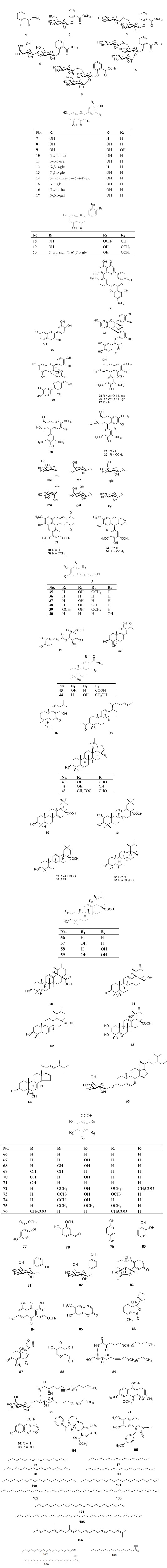
The structures of compounds **1**–**109** from *Gaultheria*

To date, extensive investigations on the chemical constituents from 34 species in the genus *Gaultheria* have led to the identification of 110 compounds, mostly assigned to five structural types, including methyl salicylate derivatives, C_6_-C_3_ constituents, terpenoids, steroids and other compounds. From an overall perspective, a majority of these compounds were found in two plants, *G*. *yunnanensis* and *G*. *nummularioides*. In this section, we summarize and classify all of the constituents in the genus *Gautheria* that have been reported. Their structures are shown below (see Figure 1), while the corresponding plant sources and references are collected in Table 1.

**Table 1:** Chemical Constituents from *Genus* *Gaultheria*.

No.	Name	Resource	Plant part	Ref.
**1**	Methyl salicylate	*G*. *yunnanensis*	Aerial part	[6]
**2**	Methyl salicylate 2-*O*-*β*-d-glucopyranoside	*G*. *yunnanensis*	Seed	[7]
**3**	Gaultherin	*G*. *yunnanensis*	Aerial part	[3,6]
		*G*. *yunnanensis*	Seed	[7]
**4**	Methyl salicylate 2-*O*-*β*-d-lactoside	*G*. *yunnanensis*	-	[8]
**5**	MSTG-A	*G*. *yunnanensis*	Aerial part	[3]
**6**	MSTG-B	*G*. *yunnanensis*	Aerial part	[3]
**7**	Kaempferol	*G*. *hispida*	Leaf	[9]
		*G*. *miqueliana*	Leaf	[9]
		*G*. *procumbens*	Leaf	[9]
		*G*. *trichophylla*	Leaf	[9]
**8**	Quercetin	*G*. *cuneata*	Leaf	[9]
		*G*. *depressa*	Leaf	[9]
		*G*. *hispida*	Leaf	[9]
		*G*. *hookeri*	Leaf	[9]
		*G*. *itoana*	Leaf	[9]
		*G*. *miqueliana*	Leaf	[9]
		*G*. *mucronata*	Leaf	[9]
		*G*. *procumbens*	Leaf	[9]
		*G*. *shallon*	Leaf	[9]
		*G*. *tasmanica*	Leaf	[9]
		*G*. *trichophylla*	Leaf	[9]
		*G*. *yunnanensis*	Roots	[10]
		*G*. *nummularioides*	Whole plant	[11]
**9**	Myricetin	*G*. *yunnanensis*	Seed	[7]
		*G*. *shallon*	Leaf	[9]
**10**	Quercitrin	*G*. *yunnanensis*	Whole plant	[10]
		*G*. *nummularioides*	Whole plant	[11]
**11**	Avicularin	*G*. *nummularioides*	Whole plant	[11]
**12**	Kaempferol-3-*O*-*β*-d-glucuronide	*G*. *leucocarpa* var. *yunnanensis* or var. *crenulata*	Aerial part	[6]
**13**	Quercetin-3-*O*-*β*-d-glucuronide	*G*. *leucocarpa* var. *yunnanensis* or var. *crenulata*	Aerial part	[6]
		*G*. *miqueliana*	Leaf	[12]
**14**	Rutin	*G*. *yunnanensis*	Root	[10]
**15**	Hyperin	*G*. *nummularioides*	Whole plant	[13]
**16**	Quercetin-3-*O*-*α*-l-rhamnopyranoside	*G*. *nummularioides*	Whole plant	[13]
**17**	Quercetin 3-galactoside	*G*. *fragrantissima*	Leaf	[14]
**18**	(+)-Homoeriodictyol	*G*. *nummularioides*	Whole plant	[11]
**19**	Hesperetin	*G*. *nummularioides*	Whole plant	[11]
**20**	Hesperidin	*G*. *nummularioides*	Whole plant	[11]
**21**	Ginkgetin	*G*. *yunnanensis*	Seed	[7]
**22**	(+)-Catechin	*G*. *yunnanensis*	Root	[10]
**23**	Proanthocyanidin A-2	*G*. *yunnanensis*	Root	[10]
**24**	Pavetannin A-1	*G*. *nummularioides*	Whole plant	[11]
**25**	(+)-Lyoniresinol-2*α*-*O*-*β*-l-arabinopyranoside	*G*. *yunnanensis*	Root	[15]
			Root, stem and leaf	[16]
		*G*. *griffithiana*	Root, stem and leaf	[16]
		*G*. *tetramera*	Root, stem and leaf	[16]
		*G*. *leucocarpa* var. *cumingiana*	Root, stem and leaf	[16]
		*G*. *fragrantissima*	Root, stem and leaf	[16]
**26**	(+)-Lyoniresinol-2*α*-*O*-*β*-d-glucopyranoside	*G*. *yunnanensis*	Root	[15]
			Root, stem and leaf	[16]
		*G*. *griffithiana*	Root, stem and leaf	[16]
		*G*. *tetramera*	Root, stem and leaf	[16]
		*G*. *leucocarpa* var. *cumingiana*	Root, stem and leaf	[16]
		*G*. *fragrantissima*	Root, stem and leaf	[16]
**27**	(+)-Lyoniresinol	*G*. *yunnanensis*	Root	[17]
**28**	(-)-5'-Methoxyisolariciresinol	*G*. *yunnanensis*	Root	[17]
**29**	(-)-Isolariciresinol-2*α*-*O*-*β*-d-xylopyranoside	*G*. *yunnanensis*	Root	[15]
			Root, stem and leaf	[16]
		*G*. *griffithiana*	Root, stem and leaf	[16]
		*G*. *tetramera*	Root, stem and leaf	[16]
		*G*. *leucocarpa* var. *cumingiana*	Root, stem and leaf	[16]
		*G*. *fragrantissima*	Root, stem and leaf	[16]
**30**	(-)-5'-Methoxyisolariciresinol-2*α*-*O*-*β*-d-xylopyranoside	*G*. *yunnanensis*	Root	[15]
**31**	Gaultherin A	*G*. *yunnanensis*	Root	[18]
		*G*. *leucocarpa* var. *yunnanensis* or var. *crenualta*	Aerial part	[6]
**32**	Gaultherin B	*G*. *yunnanensis*	Root	[18]
		*G*. *leucocarpa* var. yunnanensis or var. *crenualta*	Aerial part	[6]
**33**	Gaultherin D	*G*. *yunnanensis*	Root	[19]
**34**	Gaultherin C	*G*. *yunnanensis*	Root	[19]
**35**	Ferulic acid	*G*. *yunnanensis*	Root	[10]
		*G*. *nummularioides*	-	[20]
		*G*. *shallon*	-	[20]
		*G*. *fragrantissima*	-	[20]
		*G*. *cuneata*	-	[20]
		*G*. *griffithiana*	-	[20]
		*G*. *pyroloides*	-	[20]
		*G*. *procumbens*	-	[20]
		*G*. *depressa*	-	[20]
		*G*. *hispida*	-	[20]
		*G*. *tetramera*	-	[20]
		*G*. *thymifolia*	-	[20]
		*G*. *yunnunense*	-	[20]
		*G*. *rengifoana*	-	[20]
		*G*. *hispida*	-	[20]
		*G*. *wisleyensis*	-	[20]
		*G*. *eriophylla*	-	[20]
**36**	Cinnamic acid	*G*. *itoana*	Whole plant	[21]
		*G*. *procumbens*.	Leaf	[22]
**37**	*p*-Coumaric acid	*G*. *nummularioides*	-	[20]
		*G*. *nummularioides*	-	[20]
		*G*. *shallon*	-	[20]
		*G*. *fragrantissima*	-	[20]
		*G*. *cuneata*	-	[20]
		*G*. *griffithiana*	-	[20]
		*G*. *hookeri*	-	[20]
		*G*. *itoana*	-	[20]
		*G*. *pyroloides*	-	[20]
		*G*. *procumbens*	-	[20]
			Leaf	[23]
		*G*. *depressa*	-	[20]
		*G*. *tetramera*	-	[20]
		*G*. *thymifolia*	-	[20]
		*G*. *yunnunense*	-	[20]
		*G*. *rengifoana*	-	[20]
		*G*. *hispida*	-	[20]
		*G*. *wisleyensis*	-	[20]
		*G*. *eriophylla*	-	[20]
		*G*. *procumbens*.	Leaf	[22]
**38**	Caffeic acid	*G*. *nummularioides*	-	[20]
		*G*. *nummularioides*	-	[20]
		*G*. *shallon*	-	[20]
		*G*. *adenothrix*	-	[20]
		*G*. *ovatifolia*	-	[20]
		*G*. *humifusa*	-	[20]
		*G*. *fragrantissima*	-	[20]
		*G*. *cuneata*	-	[20]
		*G*. *griffithiana*	-	[20]
		*G*. *hookeri*	-	[20]
		*G*. *itoana*	-	[20]
		*G*. *pyroloides*	-	[20]
		*G*. *procumbens*	-	[20,24]
			Leaf	[23]
		*G*. *depressa*	-	[20]
		*G*. *hispida*	-	[20]
		*G*. *tetramera*	-	[20]
		*G*. *thymifolia*	-	[20]
		*G*. *yunnunense*	-	[20]
		*G*. *rengifoana*	-	[20]
		*G*. *hispida*	-	[20]
		*G*. *wisleyensis*	-	[20]
		*G*. *eriophylla*	-	[20]
		*G*. *procumbens*.	Leaf	[22]
**39**	Sinapic acid	*G*. *nummularioides*	-	[20]
		*G*. *shallon*	-	[20]
		*G*. *griffithiana*	-	[20]
		*G*. *itoana*	-	[20]
		*G*. *hispida*	-	[20]
		*G*. *tetramera*	-	[20]
		*G*. *thymifolia*	-	[20]
		*G*. *rengifoana*	-	[20]
		*G*. *wisleyensis*	-	[20]
		*G*. *eriophylla*	-	[20]
**40**	*o*-Coumaric acid	*G*. *procumbens*	Leaf	[23]
**41**	Chlorogenic acid	*G*. *yunnanensis*	Root	[10]
**42**	Gaultheronoterpene	*G*. *yunnanensis*	Root	[25]
**43**	Gaultheric acid	*G*. *yunnanensis*	Root	[25]
**44**	13-Acetyl-14,18-dihydroxy-podocarpa-8,11,13-triene	*G*. *itoana*	Whole plant	[21]
		*G*. *itoana*	Whole plant	[21]
**45**	14,18-Dihydroxyabieta-8,11,13-trien-7-one	*G*. *itoana*	Whole plant	[21]
**46**	3*β*-Acetyl-12,25-diene-dammarane	*G*. *yunnanensis*	Root	[26]
**47**	3*β*-Hydroxy-20(29)-lupen-28-aldehyde	*G*. *yunnanensis*	Root	[26]
**48**	Lupeol	*G*. *paniculata*	Aerial part	[27]
**49**	3*β*-Acetoxy-20(29)-lupen-28-aldehyde	*G*. *yunnanensis*	Root	[26]
**50**	Taraxerol	*G*. *nummularioides*	Whole plant	[11]
**51**	Maslinsaeure	*G*. *nummularioides*	Whole plant	[11]
**52**	3*β*-Acetyloleanolic acid	*G*. *yunnanensis*	Root	[26]
**53**	Oleanolic acid	*G*. *nummularioides*	Whole plant	[11]
**54**	*α*-Amyrin	*G*. *nummularioides*	Whole plant	[11]
**55**	3*β*-Acetoxy-urs-12-ene	*G*. *nummularioides*	Whole plant	[11]
**56**	Ursolic acid	*G*. *yunnanensis*	Seed	[7]
		*G*. *yunnanensis*	Root	[26]
		*G*. *subcorymbosa*	Twig	[28]
		*G*. *yunnanensis*	Aerial part	[29]
		*G*. *fragrantissima*	Leaf	[14]
		*G*. *nummularioides*	Whole plant	[13]
**57**	3*β*-Dihydroxy-urs-12-en-28-oic acid	*G*. *nummularioides*	Whole plant	[11]
**58**	Pomolic acid	*G*. *nummularioides*	Whole plant	[13]
**59**	Tormentic acid	*G*. *nummularioides*	Whole plant	[13]
**60**	Methyl ursolate	*G*. *subcorymbosa*	Twig	[28]
**61**	Uvaol	*G*. *subcorymbosa*	Twig	[28]
**62**	3*β*-Hydroxy-bauer-7-en-28-oic acid	*G*. *nummularioides*	Whole plant	[11]
**63**	Euscaphic acid	*G*. *nummularioides*	Whole plant	[11]
**64**	(22*E*,24*R*)-24-Methyl-5*α*-cholesta-7,22-diene-3*β*,5,6*β*-triol	*G*. *nummularioides*	Whole plant	[11]
**65**	Daucosterol	*G*. *yunnanensis*	Root	[7,11,17]
**66**	Benzoic acid	*G*. *miqueliana*	-	[30]
		*G*. *procumbens*.	Leaf	[31]
**67**	4-Hydroxybenzoic acid	*G*. *itoana*	Whole plant	[21]
		*G*. *nummularioides*	-	[20]
		*G*. *shallon*	-	[20]
		*G*. *fragrantissima*	-	[20]
		*G*. *cuneata*	-	[20]
		*G*. *griffithiana*	-	[20]
		*G*. *itoana*	-	[20]
		*G*. *pyroloides*	-	[20]
		*G*. *depressa*	-	[20]
		*G*. *hispida*	-	[20]
		*G*. *tetramera*	-	[20]
		*G*. *thymifolia*	-	[20]
		*G*. *rengifoana*	-	[20]
		*G*. *hispida*	-	[20]
		*G*. *wisleyensis*	-	[20]
		*G*. *procumbens*	-	[24]
		*G*. *procumbens*.	Leaf	[22]
**68**	Protocatechuic acid	*G*. *yunnanensis*	Root	[10]
		*G*. *nummularioides*	-	[20]
		*G*. *nummularioides*	-	[20]
		*G*. *shallon*	-	[20]
		*G*. *adenothrix*	-	[20]
		*G*. *ovatifolia*	-	[20]
		*G*. *humifusa*	-	[20]
		*G*. *fragrantissima*	-	[20]
		*G*. *cuneata*	-	[20]
		*G*. *griffithiana*	-	[20]
		*G*. *hookeri*	-	[20]
		*G*. *itoana*	-	[20]
		*G*. *pyroloides*	-	[20]
		*G*. *procumbens*	-	[20]
			Leaf	[23]
		*G*. *depressa*	-	[20]
		*G*. *hispida*	-	[20]
		*G*. *tetramera*	-	[20]
		*G*. *thymifolia*	-	[20]
		*G*. *rengifoana*	-	[20]
		*G*. *hispida*	-	[20]
		*G*. *wisleyensis*	-	[20]
		*G*. *eriophylla*	-	[20]
		*G*. *procumbens*.	Leaf	[22]
**69**	*o*-Pyrocatechuic acid	*G*. *nummularioides*	-	[20]
		*G*. *nummularioides*	-	[20]
		*G*. *shallon*	-	[20]
		*G*. *adenothrix*	-	[20]
		*G*. *ovatifolia*	-	[20]
		*G*. *humifusa*	-	[20]
		*G*. *fragrantissima*	-	[20]
		*G*. *cuneata*	-	[20]
		*G*. *griffithiana*	-	[20]
		*G*. *hookeri*	-	[20]
		*G*. *itoana*	-	[20]
		*G*. *pyroloides*	-	[20]
		*G*. *procumbens*	-	[20,32,33]
			Leaf	[23]
		*G*. *tetramera*	-	[20]
		*G*. *thymifolia*	-	[20]
		*G*. *yunnunense*	-	[20]
		*G*. *rengifoana*	-	[20]
		*G*. *wisleyensis*	-	[20]
		*G*. *eriophylla*	-	[20]
		*G*. *procumbens*.	Leaf	[22]
**70**	*β*-Resorcylic acid	*G*. *superba*	-	[24]
**71**	Salicylic acid	*G*. *yunnanensis*	Root	[10]
		*G*. *nummularioides*	Whole plant	[11]
		*G*. *nummularioides*	-	[20]
		*G*. *fragrantissima*	-	[20]
		*G*. *cuneata*	-	[20]
			Leaf	[9]
		*G*. *griffithiana*	-	[20]
		*G*. *hookeri*	-	[20]
			Leaf	[9]
		*G*. *itoana*	-	[20]
			Leaf	[9]
		*G*. *pyroloides*	-	[20]
		*G*. *procumbens*	-	[20]
			Leaf	[23]
			Leaf	[9]
		*G*. *hispidula*	-	[20]
		*G*. *tetramera*	-	[20]
		*G*. *rengifoana*	-	[20]
		*G*. *miqueliana*	Leaf	[34]
		*G*. *procumbens*.	Leaf	[22]
		*G*. *trichophylla*	Leaf	[9]
**72**	Acetylsyringic acid	*G*. *yunnanensis*	Root	[17]
**73**	Syringic acid	*G*. *shallon*	-	[20]
		*G*. *pyroloides*	-	[20]
		*G*. *procumbens*	-	[20]
		*G*. *hispida*	-	[20]
		*G*. *eriophylla*	-	[20]
		*G*. *procumbens*.	Leaf	[22]
**74**	Vanillic acid	*G*. *yunnanensis*	Root	[10]
		*G*. *itoana*	Whole plant	[21]
		*G*. *yunnanensis*	Aerial part	[29]
		*G*. *nummularioides*	Whole plant	[11]
		*G*. *nummularioides*	-	[20]
		*G*. *nummularioides*	-	[20]
		*G*. *shallon*	-	[20]
		*G*. *adenothrix*	-	[20]
		*G*. *humifusa*	-	[20]
		*G*. *fragrantissima*	-	[20]
		*G*. *cuneata*	-	[20]
		*G*. *griffithiana*	-	[20]
		*G*. *hookeri*	-	[20]
		*G*. *itoana*	-	[20]
		*G*. *pyroloides*	-	[20]
		*G*. *procumbens*	-	[20]
			Leaf	[23]
			-	[24]
		*G*. *depressa*	-	[20]
		*G*. *tetramera*	-	[20]
		*G*. *thymifolia*	-	[20]
		*G*. *yunnunense*	-	[20]
		*G*. *rengifoana*	-	[20]
		*G*. *hispida*	-	[20]
		*G*. *wisleyensis*	-	[20]
		*G*. *eriophylla*	-	[20]
**75**	3,4,5-Trimethoxy-benzoic acid	*G*. *yunnanensis*	Root	[17]
**76**	Gentistic acid	*G*. *yunnanensis*	Root	[10]
		*G*. *nummularioides*	-	[20]
		*G*. *nummularioides*	-	[20]
		*G*. *shallon*	-	[20]
		*G*. *adenothrix*	-	[20]
		*G*. *ovatifolia*	-	[20]
		*G*. *humifusa*	-	[20]
		*G*. *fragrantissima*	-	[20]
		*G*. *cuneata*	-	[20]
		*G*. *griffithiana*	-	[20]
		*G*. *hookeri*	-	[20]
		*G*. *itoana*	-	[20]
		*G*. *pyroloides*	-	[20]
		*G*. *procumbens*	-	[20]
			Leaf	[23]
			-	[33]
		*G*. *depressa*	-	[20]
		*G*. *hispida*	-	[20]
		*G*. *tetramera*	-	[20]
		*G*. *thymifolia*	-	[20]
		*G*. *rengifoana*	-	[20]
		*G*. *hispida*	-	[20]
		*G*. *wisleyensis*	-	[20]
		*G*. *eriophylla*	-	[20]
		*G*. *procumbens*.	Leaf	[22]
**77**	Methyl gentisate	*G*. *yunnanensis*	Aerial part	[6]
**78**	Vanillin	*G*. *procumbens*.	Leaf	[22]
**79**	Hydroquinone	*G*. *mucronata*	Leaf	[9]
**80**	(+)-Catechol	*G*. *nummularioides*	Whole plant	[11]
		*G*. *adenothrix*	-	[20]
		*G*. *ovatifolia*	-	[20]
		*G*. *humifusa*	-	[20]
**81**	Catechol-*β*-d-glucopyranoside	*G*. *ovatifolia*	Leaf	[20]
**82**	Arbutin	*G*. *adenothrix,*	Leaf	[35]
**83**	Gaultheriadiolide	*G*. *yunnanensis*	Seed	[7]
**84**	6-Ethyl-5-hydroxy-2,7-dimethoxy-1,4-naphthoquinone	*G*. *yunnanensis*	Seed	[7]
**85**	Scopoletin	*G*. *yunnanensis*	Root	[17]
**86**	Fraxinellone	*G*. *nummularioides*	Whole plant	[11]
**87**	Fraxinellonone	*G*. *nummularioides*	Whole plant	[11]
**88**	3,5-Dihydroxy-2-hydroxymethyl-4H-pyran-4-one	*G*. *leucocarpa* Bl var. *crenulata*	Leaves and stem	[36]
**89**	(2*S*,3*S*,4*R*)-*N*-[(2*R*)-Hydroxytetracosanoyl]**-**1,3,4-trihydroxy-2-amino-6-octadecene	*G*. *nummularioides*	Whole plant	[13]
**90**	(2*S*,3*S*,4*R*,8*Z*),-2-[(2*R*)-Hydroxybehenoylamino]-8-octadecene-l,3,4-triol-1-*O*-*β*-d-glucopyranoside	*G*. *nummularioides*	Whole plant	[13]
**91**	Preskimmianine	*G*. *nummularioides*	Whole plant	[11]
**92**	Dictamine	*G*. *nummularioides*	Whole plant	[11]
**93**	Confusameline	*G*. *nummularioides*	Whole plant	[11]
**94**	Hirsutine	*G*. *nummularioides*	Whole plant	[11]
**95**	Gaultherialine A	*G*. *nummularioides*	Whole plant	[11]
**96**	Pentadecane	*G*. *subcorymbosa*	Leafsurface wax	[37]
		*G*. *antipoda*	Leafsurface wax	[37]
**97**	Hexadecane	*G*. *subcorymbosa*	Leafsurface wax	[37]
		*G*. *antipoda*	Leafsurface wax	[37]
**98**	Heptadecane	*G*. *subcorymbosa*	Leafsurface wax	[37]
		*G*. *antipoda*	Leafsurface wax	[37]
**99**	Octadecane	*G*. *subcorymbosa*	Leafsurface wax	[37]
		*G*. *antipoda*	Leafsurface wax	[37]
**100**	Nonadecane	*G*. *subcorymbosa*	Leafsurface wax	[37]
		*G*. *antipoda*	Leafsurface wax	[37]
**101**	Eicosane	*G*. *subcorymbosa*	Leafsurface wax	[37]
		*G*. *antipoda*	Leafsurface wax	[37]
**102**	Heneicosane	*G*. *subcorymbosa*	Leafsurface wax	[37]
		*G*. *antipoda*	Leafsurface wax	[37]
**103**	Docosane	*G*. *subcorymbosa*	Leafsurface wax	[37]
		*G*. *antipoda*	Leafsurface wax	[37]
**104**	Tricosane	*G*. *subcorymbosa*	Leafsurface wax	[37]
		*G*. *antipoda*	Leafsurface wax	[37]
**105**	*n*-Dotriacontane	*G*. *yunnanensis*	Aerial part	[29]
**106**	Squalene	*G*. *nummularioides*	Whole plant	[11]
**107**	Octadecanol	*G*. *nummularioides*	Whole plant	[13]
**108**	Palmitic acid	*G*. *yunnanensis*	Root	[17]
		*G*. *itoana*	Whole plant	[21]
		*G*. *nummularioides*	Whole plant	[13]
**109**	Stearic acid	*G*. *itoana*	Whole plant	[21]

- no marked plant part.

### 2.1. Methyl Salicylate Derivatives

Methyl salicylate (**1**) and its glycoside derivatives **2**–**6**, namely the methyl salicylate derivatives obtained from *Gautheria* species, possess remarkable analgesic and anti-inﬂammatory activities [3,6,7,8,38,39]. Methyl salicylate (**1**) is the chief component in the ethanol extract of the aerial part of *G*. *yunnanensis*. It also represents a large percentage in the volatile oils of *Gaultheria* plants [3,6,7]. Methyl salicylate has a wide range of applications in flavors, organic synthesis intermediates and solvents [3,38]. Medically, it has an important role in the anti-inflammatory and analgesic effects for dental medicine [38,39]. Structurally, methyl salicylate 2-*O*-*β*-d-glucopyranoside (**2**), gaultherin (**3**), methyl salicylate 2-*O*-*β*-d-lactoside (**4**), methyl benzoate-2-*O*-*β*-d-xylopyranosyl(1-2)[*O*-*β*-d-xylo-pyranosyl(1-6)]-*O*-*β*-d-glucopyranoside (MSTG-A) (**5**) and methyl benzoate-2-*O*-*β*-d-glucopyranosyl-(1-2)[*O*-*β*-d-xylopyranosyl(1-6)]-*O*-*β*-d-glucopyranoside (MSTG-B) (**6**) have one glucopyranosyl unit connected by an *O*-glycosidic bond to the C(1) position in the parent salicylic acid nucleus, and the carboxylic acid group in the C(2) position contains a methyl group in a formate ester. Compound **3** has a xylopyranosyl bound to the C(6′) of compound **2**, and in compound **4** it is one galactopyranosyl. Up to now, methyl salicylate 2-*O*-*β*-d-lactoside (**4**) and MSTG-A (**5**) were only found in the *G*. *yunnanensis*. Those ingredient**s** are probably the dominant chemical constituents of the *G*. *yunnanensi*s responsible for curing rheumatism in traditional ethno-medicinal applications [3]. Nowadays many researchers consider the methyl salicylate glycosides as characteristic marker components in this genus, and as non-steroidal anti-inflammation drug (NSAID) leads, devoting themselves to finding other examples of those types of components in the genus, and trying to elucidate their mechanism of action for fighting rheumatism.

### 2.2. C_6_-C_3_ *Constituents*

There are 19 flavonoids, 10 lignans and seven simple phenylpropanoids in the genus *Gaultheria* [6,7,9,10,11,12,13,14,15,16,17,18,19,20,21,22,23,24,29].

#### 2.2.1. Flavonoids

The sorts and contents of flavonoid in *Gaultheria* are relatively fewer than that of the other genus in the family Ericaceae. It has 18 flavonoids (compounds **7**–**24**), mainly distributed in *G*. *yunnanensis* and *G*. *nummularioides* [6,7,9,10,11,12,13,14,29].

Compounds **7**–**17** are flavonols, with the C(5) and C(7) in ring-A, together with C(4′) in ring-B attached to hydroxyls. In compounds **9**–**17** the flavonol-aglycone is linked with different glycosyl groups (e.g., glucosyl, rhamnosyl, mannosyl, arabinosyl and galactosyl) at the C(3) position through *O*-glycosidic linkages. Kaempferol-3-*O*-*β*-d-glucuronide (**12**) and quercetin-3-*O*-*β*-d-glucuronide (**13**) are the only two glucuronides reported in *G*. *yunnanensis* [6]. Quercetin-3-*O*-*α*-l-rhamnopyranoside (**16**) was isolated from *G*. *nummularioides*, while hyperoside (**17**) comes from the leaves of *G*. *fragrantissima*. (+)-Homoeriodictyol (**18**), hesperetin (**19**) and hesperidin (**20**), which all were detected in the *G*. *nummularioides*, belong to the flavonone class, [11,13,14].Another, ginkgetin (**21**), is a dimeric-flavone. Proanthocyanidin A-2 (**23**) was found in the roots of *G*. *yunnanensis*, and pavetannin A-1 (**24**) in *G*. *nummularioides* [10,11].

#### 2.2.2. Lignans

Compounds **25**–**34** are cyclolignans, in which the aromatic carbon atom at the C(6) position of one phenylpropanoid unit is directly linked with the aliphatic carbon at the C(7′) position in the other C_6_-C_3_ unit. These sorts of compounds were also regarded in the past as active components against rheumatism in this genus [15,16,17,18,19]. The ten abovementioned compounds were all found in *G*. *yunnanensis* [6,15,16,17,18,19]. (+)-Lyoniresinol-2*α*-*O*-*β*-l-arabinopyranoside (**25**), (+)-lyoniresinol-2*α*-*O*-*β*-d-glucopyranoside (**26**) and (−)-isolariciresinol-2*α*-*O*-*β*-d-xylopyranoside (**29**) occur in the plants *G*. *griffithiana*, *G*. *tetramera* and *G*. *fragrantissima* [15,16,40]. The three lignans **25**, **26** and **29** do not possess anti-inflammatory effects, while the *n*-butanol fraction from the roots of *G*. *yunnanensis* is rich in these three compounds and shows outstanding anti-inflammatory activities [40]. Ma *et al.* measured the contents of these three lignans from different parts of five species, *G*. *fragrantissima*, *G*. *leucocarpa* var. *yunnanensis*, *G*. *leucocarpa* var. *cumingiana*, *G*. *tetramera* and *G*. *griffithiana*. The contents of compounds **25** and **29** are the highest in the stems and roots of *G*. *leucocarpa* var. *yunnanensis*, and the lowest in the stems of *G*. *fragrantissima*. Moreover, the content of (+)-lyoniresinol-2*α*-*O*-*β*-d-glucopyranoside (**26**) is the highest in the roots of *G*. *fragrantissima* and the lowest in the roots of *G*. *leucocarpa* var. *yunnanensis*. The former is 25 times the latter. The sequence of the total content of those three lignans in roots is *G*. *fragrantissima* > *G*. *leucocarpa* var. *yunnanensis* > *G*. *leucocarpa* var. *cumingiana* > *G*. *tetramera* > *G*. *griffithiana* [16].

5-Methoxy-(+)-isolariciresinol-9,9′-diacetate (**31**) and (+)-lyoniresinol-9,9′-diacetate (**32**) are novel cyclolignan esters with two acetyls connected with the C(9) and C(9′) positions in the two lignans. The phytochemical properties, spectral analyses and chemical degradation play important roles in the chemical structures elucidation of the two compounds [6,18]. The compounds gaultherin D (**33**) and gaultherin C (**34**) are found in the roots of *G*. *yunnanensis*, which are employed as a medicinal part by the Bai nationality living in Yunnan Province [19].

#### 2.2.3. Simple Phenylpropanoids

The seven simple phenylpropanoids **35**–**41** are phenylacrylic acid derivatives. Ferulic acid (**35**), *p*-coumaric acid (**37**), caffeic acid (**38**) and sinapic acid (**39**) are widely reported in many plants of the genus *Gaultheria* [10,20,21,22,23,24].

### 2.3. Terpenoids

There are four diterpenes and 18 triterpenoids in the genus *Gaultheria* [7,11,13,14,21,25,26,27,28,29]. Compounds **42**–**45** are diterpenes with tricyclic podocarpane-type skeletons, which were firstly reported in the genus *Gaultheria*. As new diterpenes, they might be useful as chemotaxonomic markers [21,25]. Among them, gaultheronoterpene (**42**) and gaultheric acid (**43**) are wildly distributed in the roots of *G*. yunnanensis [25].

Eighteen triterpenoids (from **46** to **63**) include one dammarane tetracyclic triterpene, as well as lupane, oleanane, and ursane pentacyclic triterpanes. Among them, 3*β*-acetyl-dammarane-12,25-diene (**46**) was the first dammarane-type compound discovered in the family Ericaceae. Compounds **47**–**49** are lupine triterpenoids. Both **47** and **48** display a hydroxyl functional-group at the C(3) position, while **49** has an acetoxy group instead. The methyl is joined to C(28) in compounds **48** and **49**, and it is replaced by an aldehyde group in **47**. As for the four oleanane-triterpenoids, taraxerol (**50**), maslinsaeure (**51**), 3*β*-acetyloleanoic acid (**52**) and oleanolic acid (**53**), the C-17 position is attached to a methyl (C-28) in **50**, while in **51**–**53** it has a carboxyl (C-28). The C-3 location of **52** is an acetoxy group, and the other oleanane-type triterpenoids have a hydroxyl in the same place. Compounds **54**–**63** belong to the *α*-amyrane type triterpenoids. Ursolic acid (**56**) from *G*. *yunnanensis*, *G*. *subcorymbosa, G*. *fragrantissima* and *G*. *nummularioides* is used in the cosmetics industry [7,11,13,14,26,28,29,41]. Compounds **60** and **61** were obtained from the twigs of *G*. subcorymbosa, and the plant *G*. *nummularioides* contains compounds **54**, **55**, **57**, **58** **59**, **62** and **63** [11,13,28].

### 2.4. Steroids

Only two steroids were reported in the genus *Gaultheria*. They are (22*E*,24*R*)-24-methyl-5*α*-cholesta-7,22-diene-3*β*,5*α*,6*β*-triol (**64**) and daucosterol (**65**), obtained from *G*. *yunnanensis* and *G*. *nummularioides*, respectively [7,11,17].

### 2.5. Other Compounds

Compounds **66**–**109**, which include benzoic acid derivatives, alkaloids, anthraquinones, dilactones and hydrocarbons, were obtained from *G*. *yunnanensis*, *G*. *nummularioides*, *G*. *shallon*, *G*. *adenothrix*, *G*. *ovatifolia*, *G*. *humifusa*, *G*. *fragrantissima*, *G*. *cuneata*, *G*. *griffithiana*, *G*. *hookeri*, *G*. *itoana* and *G*. *pyroloides* [6,7,9,10,11,13,17,20,21,22,23,24,29,30,31,32,33,34,35,36,37].

Compounds **66**–**77** are benzoic acid derivatives, with several hydroxyl-, methoxyl- and formoxyl- groups connected to different positions of the benzoic acid. Methyl gentisate (**77**) from the aerial part of *G*. *yunnanensis*, is usually used as a skin-lightener and antioxidant. It appears to be more efficient than the free acid as well as other well-known hypopigmentation agents [6,42].

Gaultheriadiolide (**83**), a new dilactone from the seeds of *G*. *yunnanensis*, exhibited medium cytotoxic effect against HEp-2 and HepG2 cells, with IC_50_ of 23.337 μM and 29.4497 μM, respectively [43]. 6-Ethyl-5-hydroxy-2,7-dimethoxy-1,4-naphthoquinone (**84**) in the seeds of *G*. *yunnanensis*, was the only reported anthraquinone in the genus *Gaultheria* [7]. 

Fraxinellone (**86**) and fraxinellonone (**87**) are degradation products of the limonoids in *G*. *nummularioides*, which have obvious pharmacological activities [11]. Compound **86** possessed neuroprotective and vasorelaxing effects [17,44]. Fraxinellonone (**87**) exhibited moderate insect-antifeeding activity and ichthyotoxicity [45]. 3,5-Dihydroxy-2-hydroxymethyl-4H-pyran-4-one (**88**), is referred to as 3-hydroxykojic acid and 3-oxykojic acid. Structurally speaking, as the asymmetric unit, it consists of two nearly parallel molecules connected with a strong intermolecular O—H^…^O hydrogen bond. This compound is only derived from *G*. *leucocarpa*, and is used for the treatment of rheumatoid arthritis, swelling pain, trauma, chronic tracheitis, cold and vertigo [36].

The alkaloid compounds **89**–**95** were only found in *G*. *nummularioides* [11,13]. They possess several novel structural features, in which (2*S*,3*S*,4*R*,8*Z*),-2-[(2*R*)-hydroxybehenoylamino]-8-octadecene-l,3,4-triol-1-*O*-*β*-d-glucopyranoside (**90**) is the mono-glycoside of compound **89**. Preskimmianine (**91**), dictamine (**92**), and confusameline (**93**) are quinoline derivatives. Hirsutine (**94**) is an indole alkaloid and gaultherialine A (**95**) was reported in the genus *Gaultheria* as a novel alkaloid [11].

Compounds **96**–**105** are all alkanes having no branched chains, and they are derived from the leaf surface waxes of *G*. *subcorymbosa* and *G*. *antipoda* in addition to *n*-dotriacontane (**105**) [29,37]. Squalene (**106**) and octadecanol (**107**) occur in *G*. *nummularioides*. Palmitic acid (**108**) and stearic acid (**109**) are two saturated fatty acids found in the genus *Gaultheria* [13,17,21].

## 3. Volatile Chemical Constituents

The plants of the genus *Gaultheria* were first studied for their aromatic character. The essential oils of this genus were usually obtained by hydrodistillation, and their structures elucidated by gas chromatography-mass spectrometry (GC-MS) or solid-phase micro-extraction gas chromatography-mass spectrometry (SPME-GC-MS) [43,46,47,48,49,50,51,52,53,54]. Ninety seven chemical constituents were recently reported from the essential oils of four *Gaultheria* plants: *G*. *yunnanensis*, *G*. *leucocarpa* Bl var. *crenulata*, *G*. *fragrantissima* and *G*. *procumbens* (see Table 2). These essential oils are composed primarily of methyl salicylate (five compounds), alkanes (19 compounds), monoterpenes (22 compounds), sesquiterpenes (14 compounds) and aromatic derivatives (nine compounds). Methyl salicylates are major components found at fairly high concentrations (70–99%) in contrast with other components present in only trace amounts. Through odor-evaluation and blending, it was determined that essential oils of *G*. *yunnanensis* a sweet and long staying, and thus more suitable to make gum essence and tooth-paste fragrances. They have been the subject of extensive studies due to their economic importance. The constituents showed good prospects for application in the fragrance industry [47].

## 4. Biological Activities

Many studies have verified that the extracts and compounds derived from *Gaultheria* plants exhibit a wide spectrum of pharmacological activities *in vitro* and *in vivo*, covering anti-inﬂammatory, analgesic, anti-oxidative and antibacterial properties. 

### 4.1. Anti-Inﬂammatory Activities

It was found that the H_2_O, EtOAc and *n*-butanol extracts of *G*. *leucocarpa* had remarkable anti-inflammatory activity by significantly reducing the level of joint swelling in a rat adjuvant-induced arthritis model [40].

A salicylate derivative fraction (SDF), which is rich in gaultherin (**3**) reported from *G*. *yunnanensis*, exhibited a significant inhibition of pain and inflammatory processes. Beyond that, compared with indomethacin, a positive control, SDF has strong inhibitory activity on the hind paw edema (200, 400 mg/kg body wt., *p*.*o*.) and ear swelling tests in mice (200, 400, 800 mg/kg body wt., *p*.*o*.) caused by carrageen and croton oil, respectively [3].

**Table 2:** Essential Oils of *Genus* *Gaultheria*.

No.	Name	Source	Plant part	Percentage (%)	Ref.
**1**	Methyl salicylate	*G*. *yunnanensis*	Stem and leaf	98.85	[43]
		*G*. *yunnanensis*	Rhizome	74.18	[46]
		*G*. *leucocarpa* Bl var. *crenulata*	Leaf	95.93	[47]
		*G*. *yunnanensis*	Stem, Leaf, Root	99.66	[48]
		*G*. *yunnanensis*	Whole plant	99.66	[49]
		*G*. *yunnanensis*	Root	89.82	[50]
		*G*. *yunnanensis*	Branch and leaf	99.62	[51]
		*G. fragrantissima*	Leaf	94.60	[52]
		*G. fragrantissima*	Leaf	97.00	[53]
		*G. procumbens*	-	96.90	[54]
**2**	Ethyl salicylate	*G*. *yunnanensis*	Stem and leaf	0.05	[43]
		*G*. *leucocarpa* Bl var. *crenulata*	Leaf	0.34	[47]
		*G*. *yunnanensis*	Branch and leaf	0.02	[51]
		*G. fragrantissima*	Leaf	5.36	[52]
**3**	Phenyl salicylate	*G*. *yunnanensis*	Stem and leaf	0.11	[43]
**4**	4-Methylene-1-( *cis*)-methyl ethyl-bicyclic(3,1,0)-normal Hexane	*G*. *yunnanensis*	Stem and leaf	0.17	[43]
**5**	2-Methyl-decane	*G*. *yunnanensis*	Rhizome	1.02	[46]
**6**	Tridecane	*G*. *yunnanensis*	Rhizome	0.24	[46]
**7**	Tetradecane	*G*. *yunnanensis*	Rhizome	0.37	[46]
**8**	Pentadecane	*G*. *yunnanensis*	Rhizome	1.38	[46]
**9**	Hexadecane	*G*. *yunnanensis*	Rhizome	1.59	[46]
**10**	4-Methyl-pentadecane	*G*. *yunnanensis*	Rhizome	0.24	[46]
**11**	Heptadecane	*G*. *yunnanensis*	Rhizome	0.93	[46]
**12**	2-Methyl-hexadecane	*G*. *yunnanensis*	Rhizome	0.20	[46]
**13**	3-Methyl-hexadecane	*G*. *yunnanensis*	Rhizome	0.24	[46]
**14**	Octadecane	*G*. *yunnanensis*	Rhizome	0.27	[46]
**15**	2,6,10,14-Teramethyl-pentadecane	*G*. *yunnanensis*	Rhizome	1.04	[46]
**16**	Eicosane	*G*. *yunnanensis*	Rhizome	0.28	[46]
**17**	Phytane	*G*. *yunnanensis*	Rhizome	0.29	[46]
**18**	1-Ethyl-2-methyl cyclododecane	*G*. *yunnanensis*	Rhizome	0.30	[46]
**19**	Pregnane	*G*. *yunnanensis*	Rhizome	0.19	[46]
**20**	Methylcyclopentane	*G*. *yunnanensis*	Root	6.53	[50]
**21**	Cyclohexane	*G*. *yunnanensis*	Root	2.69	[50]
**22**	1,8-Cineole	*G*. *leucocarpa* Bl var. *crenulata*	Leaf	1.40	[47]
		*G*. *yunnanensis*	Branch and leaf	0.09	[51]
**23**	Bornyl acetate	*G*. *leucocarpa* Bl var. *crenulata*	Leaf	0.01	[49]
		*G*. *yunnanensis*	Branch and leaf	0.07	[51]
**24**	Cedrol	*G*. *yunnanensis*	Stem, Leaf, Root	trace amount	[48]
		*G*. *yunnanensis*	Whole plant	trace amount	[49]
**25**	2-Methyl-5-(1,5-dimethyl-4-hexenyl)-1,3-cyclohexadiene	*G*. *yunnanensis*	Stem and leaf	trace amount	[43]
**26**	*β*-Caryophyllene	*G*. *yunnanensis*	Rhizome	0.56	[46]
		*G*. *leucocarpa* Bl var. *crenulata*	Leaf	0.01	[47]
**27**	*β*-Maaliene	*G*. *yunnanensis*	Rhizome	0.28	[46]
**28**	Calarene	*G*. *yunnanensis*	Rhizome	1.00	[46]
**29**	*α*-Humulene	*G*. *yunnanensis*	Rhizome	0.40	[46]
		*G*. *fragrantissima*	Leaf		[53]
**30**	Germacrene D	*G*. *yunnanensis*	Rhizome	0.19	[46]
**31**	*cis*-*α*-Bisabolene	*G*. *yunnanensis*	Rhizome	0.30	[46]
**32**	*β*-Bisabolene	*G*. *yunnanensis*	Rhizome	0.38	[46]
**33**	7- *Epi*-*α*-selinene	*G*. *yunnanensis*	Rhizome	0.12	[46]
**34**	2-Hydroxy-4-methoxyacetophenone	*G*. *yunnanensis*	Rhizome	1.09	[46]
**35**	4-Methyl-2,6-ditertbutylphenol	*G*. *yunnanensis*	Rhizome	0.15	[46]
**36**	Elemicine	*G*. *yunnanensis*	Stem, Leaf, Root	trace amount	[48]
		*G*. *yunnanensis*	Whole plant	trace amount	[49]
**37**	*m*-Cymene	*G*. *yunnanensis*	Branch and leaf	0.04	[51]
**38**	1-Hexadecene	*G*. *yunnanensis*	Rhizome	0.13	[46]
**39**	Nonanal	*G*. *yunnanensis*	Stem and leaf	0.12	[43]
**40**	2-Decenal	*G*. *yunnanensis*	Stem and leaf	0.05	[43]
**41**	6-Methyl-5-heptene-2-one	*G*. *yunnanensis*	Stem and leaf	0.26	[43]
**42**	6,10-Dimethyl-5,9-undecadien-2-one	*G*. *yunnanensis*	Stem and leaf	0.29	[43]
**43**	Ethyl laurate	*G*. *yunnanensis*	Rhizome	0.42	[46]
**44**	Ethyl myristate	*G*. *yunnanensis*	Rhizome	0.60	[46]
**45**	Bornane-2,6-dione	*G*. *yunnanensis*	Rhizome	0.21	[46]
**46**	Ethyl pentadecanoate	*G*. *yunnanensis*	Rhizome	0.16	[46]
**47**	Ethyl palmitate	*G*. *yunnanensis*	Rhizome	0.43	[46]
**48**	Driman-3-ol	*G*. *yunnanensis*	Rhizome	0.18	[46]
**49**	Hexanol	*G*. *yunnanensis*	Stem, Leaf, Root	0.03	[48]
		*G*. *yunnanensis*	Whole plant	0.03	[49]
**50**	Hexanal	*G*. *yunnanensis*	Stem, Leaf, Root	0.03	[48]
**51**	*trans*-2-Hexenal	*G*. *yunnanensis*	Stem, Leaf, Root	0.16	[48]
**52**	Hexenal	*G*. *yunnanensis*	Whole plant	0.03	[49]
**53**	Palmitic	*G*. *yunnanensis*	Root	0.18	[50]
**54**	9-Octadecenic acid	*G*. *yunnanensis*	Root	0.04	[50]
**55**	Hexaacetyl-mannitol	*G*. *yunnanensis*	Root	0.04	[50]
**56**	Sorbitol-hexaacetate	*G*. *yunnanensis*	Root	0.03	[50]
**57**	Benzyl salicylate	*G*. *leucocarpa* Bl var. *crenulata*	Leaf	0.07	[47]
**58**	*p*-Hydroxy-methyl salicylate	*G*. *leucocarpa* Bl var. *crenulata*	Leaf	0.04	[47]
**59**	l,3,3-Trimethyl-tricyclo[2,2,1,O^2,6^]-heptane	*G*. *leucocarpa* Bl var. *crenulata*	Leaf	0.14	[47]
**60**	Linalool	*G*. *leucocarpa* Bl var. *crenulata*	Leaf	0.03	[47]
**61**	Geraniol	*G*. *leucocarpa* Bl var. *crenulata*	Leaf	0.10	[47]
**62**	Citronellal	*G*. *leucocarpa* Bl var. *crenulata*	Leaf	0.05	[47]
**63**	Methyl geranate	*G*. *leucocarpa* Bl var. *crenulata*	Leaf	0.01	[47]
**64**	Neral	*G*. *leucocarpa* Bl var. *crenulata*	Leaf	0.01	[47]
**65**	*α*-Thujene	*G*. *leucocarpa* Bl var. *crenulata*	Leaf	0.02	[47]
**66**	*p*-Mentha-1(7),2-diene	*G*. *leucocarpa* Bl var. *crenulata*	Leaf	0.10	[47]
**67**	Carane	*G*. *leucocarpa* Bl var. *crenulata*	Leaf	0.01	[47]
**68**	*α*-phellandrene	*G*. *leucocarpa* Bl var. *crenulata*	Leaf	0.03	[47]
**69**	*β*-Elemene	*G*. *leucocarpa* Bl var. *crenulata*	Leaf	0.06	[47]
**70**	*α*-Terpinene	*G*. *leucocarpa* Bl var. *crenulata*	Leaf	0.01	[47]
**71**	1,8(9)-p-Menthadiene	*G*. *leucocarpa* Bl var. *crenulata*	Leaf	0.01	[47]
**72**	Myrcene	*G*. *leucocarpa* Bl var. *crenulata*	Leaf	0.18	[47]
		*G*. *procumbens*	-	0.09	[54]
**73**	Ocimene	*G*. *leucocarpa* Bl var. *crenulata*	Leaf	0.58	[47]
**74**	*α*-Pinene	*G*. *leucocarpa* Bl var. *crenulata*	Leaf	0.01	[47]
		*G*. *fragrantissima*	Leaf	trace amount	[53]
		*G*. *procumbens*	-	0.22	[54]
**75**	*β*-Pinene	*G*. *leucocarpa* Bl var. *crenulata*	Leaf	0.13	[47]
		*G*. *fragrantissima*	Leaf	trace amount	[53]
		*G*. *procumbens*	-	0.25	[54]
**76**	Isoeugenol	*G*. *leucocarpa* Bl var. *crenulata*	Leaf	0.01	[47]
**77**	Methyl isoeugenol	*G*. *leucocarpa* Bl var. *crenulata*	Leaf	0.01	[47]
**78**	Aromadendrene	*G*. *leucocarpa* Bl var. *crenulata*	Leaf	0.01	[47]
**79**	2,3,5,6-Tetramethyl- *p*-benzoquirione	*G*. *leucocarpa* Bl var. *crenulata*	Leaf	0.15	[47]
**80**	1,3,5-Trimcthyl-2-methoxy-benzene	*G*. *leucocarpa* Bl var. *crenulata*	Leaf	0.01	[47]
**81**	2,6-Dithydoxy-benzoicacid methylester	*G*. *leucocarpa* Bl var. *crenulata*	Leaf	0.02	[47]
**82**	Eugenol	*G*. *leucocarpa* Bl var. *crenulata*	Leaf	0.02	[47]
**83**	Methyl eugenol	*G*. *leucocarpa* Bl var. *crenulata*	Leaf	0.01	[47]
**84**	1-Undecene	*G*. *leucocarpa* Bl var. *crenulata*	Leaf	0.01	[47]
**85**	3-Methyl-2-butanol	*G*. *leucocarpa* Bl var. *crenulata*	Leaf	0.03	[47]
**86**	3-Hexen-1-ol	*G*. *leucocarpa* Bl var. *crenulata*	Leaf	0.15	[47]
**87**	*γ*-Ionone	*G*. *leucocarpa* Bl var. *crenulata*	Leaf	0.01	[47]
**88**	4-Acetoxy-1- *p*-menthene	*G*. *leucocarpa* Bl var. *crenulata*	Leaf	0.02	[47]
**89**	Geranyl acetate	*G*. *leucocarpa* Bl var. *crenulata*	Leaf	0.01	[47]
**90**	6-Methyl-1-heptanol	*G*. *leucocarpa* Bl var. *crenulata*	Leaf	0.01	[47]
**91**	△3-Carene	*G*. *fragrantissima*	Leaf	trace amount	[53]
**92**	Longifolene	*G*. *fragrantissima*	Leaf	0.80	[53]
**93**	Caryophylene oxide	*G*. *fragrantissima*	Leaf	trace amount	[53]
**94**	Limonene	*G*. *procumbens*	-	2.17	[54]
**95**	Sabinene	*G*. *procumbens*	-	0.08	[54]
**96**	Fenchone	*G*. *procumbens*	-	0.17	[54]
**97**	Menthone	*G*. *procumbens*	-	0.12	[54]

-no marked plant part.

Gaultherin (**3**), having a similar chemical structure to aspirin, inhibited the abdominal contractions in the acetic acid-induced writhing test in mice at a dosage of 200 mg/kg. Compared to aspirin, it did not show gastric ulcerogenic effects, which is the main clinical side-effect of aspirin. The possible reason is that gaultherin released salicylate in the intestine slowly, not in stomach and it left the cyclooxygenase-1 unaffected. It was the source of cytoprotective prostaglandin in gastric epithelium [55]. Methyl salicylate 2-*O*-*β*-d-lactoside (**4**) inhibits the IKK/NF-κB signal pathway to protect from LPS-induced inflammation [8]. MSTG-A (**5**) and MSTG-B (**6**) from *G*. *yunnanensis* display anti-inflammatory effects through inhibiting the production of pro-inflammatory cytokines, NO and ROS. The two methyl salicylate glycosides dose-dependently inhibited the production of tumor necrosis factor-α (TNF-α), interleukin-1β (IL-1β), and IL-6, respectively. They also can remarkably suppress the accumulation of NO, with an inhibitory rate of 56.20% and 51.72% at 3.0 μg/mL concentration, respectively [56].

### 4.2. Analgesic Activities

Zhang *et* *al.* reported that EtOAc and *n*-butanol fractions of *G*. *yunnanensis* roots (100 mg/kg and 200 mg/kg) have remarkable anti-inflammatory effects through significantly inhibiting murine peritoneal capillary permeability [57].

### 4.3. Anti-Oxidative Activities

Li *et al* analyzed the antioxidant capacity of different polar parts and furthermore, gradient elution samples obtained through macroporous resin column chromatography from an EtOH extract of *G*. *leucocarpa* were also tested. The ethyl acetate part and the 100% MeOH-elution part showed more striking ABTS and DPPH radical scavenging effects. The major constituent of the 100% MeOH is quercetin-3-*O*-*β*-d-glucuronide (**13**), which is suggested to be responsible for the efficacy [58]. In 2011, it was demonstrated that the fruit extracts of *G*. *fragrantissima* and *G*. *tiliaefolia* possessed anti-oxidative activities [59]. The EtOAc extract of *G*. *shallon* displayed a high anti-oxidative activity in scavenging DPPH with an IC_50_ value of 14.76 ± 0.85 μg/mL, compared to ascorbic acid (IC_50_ = 18.53 ± 1.58 µg/mL), as reference compound [60].

### 4.4. Antibacterial Activities

The 95% EtOH extract, the EtOAc, and *n*-butanol fractions of the stems or roots of *G*. *leucocarpa* significantly inhibited *Staphylococcus aureus*. Additionally, the EtOAc and *n*-butanol parts of *G*. *leucocarpa* stem revealed certain inhibitory effects towards *Escherichia coli* and *Pseudomonas aeruginosa* [61]. The essential oil from the leaves of *G*. *yunnanens* presented similar antibacterial effects as methyl salicylate. It has antibacterial activity against *E*. * coli* and *S*. *aureus*, but the essential oil is superior to methyl salicylate, and the lowest antimicrobial concentration is 0.3125% and 5%, respectively [38].

### 4.5. Others

13-Acetyl-14,18-dihydroxy-podocarpa-8,11,13-triene (**44**) and 14,18-dihydroxyabieta-8,11,13-trien-7-one (**45**) from *G*. *itoana* showed significant cytotoxicities against LNCaP. Compared with the relevant clinical chemotherapeutic drug taxol, compound **44** seemed to have lower IC_50_ value against LNCaP [21]. Gaultheriadiolide (**83**), a new dilactone from the seeds of *G*. *yunnanensis*, exhibited medium cytotoxic effect against HEp-2 and HepG2 cells, with IC_50_ of 23.337 μM and 29.4497 μM, respectively [43]. Fraxinellone (**86**) was reported to possess neuroprotective and vasorelaxing activities [17,44]. 

## 5. Conclusions

This article summarized a total of 109 compounds and abundant volatile components that have been reported from the genus *Gaultheria*, with 63 references cited. The genus *Gaultheria* is widely distributed all over the World, and many species have been used as traditional herbal medicines [62,63]. So far, phytochemical research on the genus has revealed the extensive presence of methyl salicylate derivatives, C_6_-C_3_ constituents, terpenoids, and other compound types, together with prolific essential oils. The pharmacological activities of pure compounds and crude extract from this genus were mainly focused on anti-inﬂammatory and analgesic properties. For their significantly anti-inﬂammatory activities, methyl salicylate glycoside is a research hotspot in the abovementioned plants. So far, some experiments point out the anti-rheumatic effects of methyl salicylate derivatives may be due to a new mechanism of action. As a whole, the phytochemical and biological investigations were mainly concentrated on the *G*. *yunnanensis*, with little or no attention being paid to other species. This species has several fractions with demonstrated anti-inﬂammatory and analgesic abilities. In view of this background, plenty of further studies are necessary in order to examine the other plants of the *Gaultheria* genus, together with the some fractions and different constituents of the *G*. *yunnanensis* to identify the medicine effects. The authors hope this review will provide valuable data for the exploration and advanced research on *Gaultheria* species.

## References

[1] Mabberiey D.J. (1997). The Plant-Book.

[2] Simon J.E., Chadwick A.F., Craker L.E. (1984). Herbs: An Indexed Bibliography.

[3] Zhang B., Li J.B., Zhang D.M., Ding Y., Du G.H. (2007). Analgesic and anti-inflammatory activities of a fraction rich in Gaultherin isolated from *Gaultheria yunnanensis* (Franch.) Rehder. Biol. Pharm. Bull..

[4] Hirsh J.M.D. (1985). Progress review: The relationship between dose of Aspirin, side-effects and antithrombotic effectiveness. Stroke.

[5] Levy M. (1974). Aspirin use in patients with major upper gastrointestinal bleeding and peptic-ulcer disease. N. Engl. J. Med..

[6] She G.M., Li D.C., Zhang Y., Guo Z.Q., Lv H.N., She D.M. (2010). Chemical consitituent of aerial parts of Dianbaizhu (*Gaultheria leucocarpa* var. *yunnanensis* or var. *crenulata*). J. Beijing Univ. Tradit. Chin. Med..

[7] Li J., Li F., Lu Y.Y., Su X.J., Huang C.P., Lu X.W. (2010). A new dilactone from the seeds of *Gaultheria yunnanensis*. Fitoterapia.

[8] Zhang T.T., Sun L., Liu R., Zhang D., Lan X., Huang C., Xin W.Y., Wang C., Zhang D.M., Du G.H. (2012). A novel naturally occurring salicylic acid analogue acts as an anti-inflammatory agent by inhibiting nuclear factor-kappaB activity in RAW264.7 macrophages. Mol. Pharm..

[9] David J., Middleton F.L.S. (1992). A chemotaxonomic survey of flavonoids and simple phenols in the leaves of *Gaultheria* L. and related genera (Ericaceae). Bot. J. Linn. Soc..

[10] Zhang Z.Z., Koike K., Guo D.A., Li C.L., Zheng J.H., Jia Z.H., Nikaido T. (1999). Studies on the chemical constituents of yunnan wintergreen root *Gaulheria yunnanensis* (II). Chin. Tradit. Herb. Drugs..

[11] Yang M.F., Li Y.Y., Li B.G., Zhang G.L. (2007). A novel alkaloid from *Gaultheria nummularioides*. J. Asian. Nat. Prod. Res..

[12] Sasaki T., Watanabe Y. (1956). Components of the leaves of *Gaultheria miqueliana* Takeda. J. Pharm. Soc. Jpn..

[13] Yang M.F. (2004). Phytochemical investigation on three plants and Studies on synthesis and characterization of mesoporous silica materials. PhD thesis.

[14] Murthy K.S., Babu M.R. (1972). Chemical investigation of the leaves of *Gaultheria fragrantissima* Wall. Indian J. Pharm..

[15] Zhang Z.Z., Guo D.A., Li C.L., Zheng J.H., Koike K., Jia Z.H., Nikaido T. (1999). Studies on the lignan glycosides from *Gaultheria yunnanensis*. Acta. Pharm. Sin..

[16] Ma X.J., Zhao L., Han Z.T., Zheng J.H., Chen X.Z. (2002). Comparison of the contents of lignans in 5 medicinal plants of *Gaultheia* by HPLC. J. Plant Resour. Environ..

[17] Zhang Z.Z., Guo D.A., Li C.L., Zheng J.H., Koike K., Jia Z.H., Nikaido T. (1998). Studies on the chemical constituents of yunnan wintergreen (*Gaultheria yuannanensis*) (I). Chin. Tradit. Herb. Drugs.

[18] Zhang Z.Z., Guo D.A., Li C.L., Zheng J.H., Koike K., Jia Z.H., Nikaido T. (1999). Gaultherins A and B, two lignans from *Gaultheria yunnanensis*. Phytochemistry.

[19] Zhang Z.Z., Guo D.A., Li C.L., Zheng J.H., Koike K., Jia Z.H., Nikaido T. (2000). Gaultherins C and D, two new lignans from the roots of *Gaultheria yunnanensis*. Heterocycles.

[20] Towers G.H.N., Tse A., Maass W.S.G. (1966). Phenolic acids and phenolic glycosides of *Gaultheria* species. Phytochemistry.

[21] Chen C.Y., Lin R.J., Huang J.C., Wu Y.H., Cheng M.J., Hung H.C., Lo W.L. (2009). Chemical constituents from the whole plant of *Gaultheria itoana* Hayata. Chem. Biodivers..

[22] Grisebach H., Vollmer K.O. (1964). On the biosynthesis of benzoic acids in *Gaultheria procumbens* L. II. Z. Naturforsch. B..

[23] EL-Basyouni S.Z., Chen D., Ibraim R.K., Neish A.C., Towers G.H.N. (1964). The biosynthesis of hydroxybenzoic acids in higher plants. Phytochemistry.

[24] Ibrahim R.K., Towers G.H.N. (1960). The identification, by chromatography, of plant phenolic acids. Arch. Biochem. Biophys..

[25] Zhang Z.Z., Guo D.A., Li C.L., Zheng J.H., Koike K., Jia Z.H., Nikaido T. (1999). Two diterpenoids from the roots of *Gaultheria yunnanensis.*. J. Nat. Prod..

[26] Zhang Z.Z., Guo D.A., Li C.L., Zheng J.H., Koike K., Jia Z.H., Nikaido T. (1999). Studies on the chemical constituents of yunnan wintergreen (*Gaultheria yuannanensis*)(III). Chin. Tradit. Herb. Drugs.

[27] Cambie R.C., Parnell J.C. (1969). A New Zealand phytochemical survey, VII. Constituents of some dicotyledons. New Zealand Journal of Science.

[28] Alauddin M., Bryce T.A., Clayton E., Martin-Smith M., Subramanian G. (1965). Triterpenoids from new zealand plants. isolation of ursolic acid from *Gaultheria subcorymbosa* Col. J. Chem. Soc..

[29] Ma X.J., Du C.F., Zheng J.H., Chen X.Z. (2001). Studies on chemical constituents of *Gaultheria leucocarpa* var. yunnanensis (Franch.) T. Z. Hsu & R. C. Fang. Chin. J. Chin. Ma. Med..

[30] Belousova M.V., Grakhov V.P., Berozovskaya T.P., Dmitruk S.U., Komissarenko N.F. (1999). Free phenolic acids of ericaceous plants of Siberia and the Russian Far East. Rastitel'Nye Resursy.

[31] Vollmer K.O., Grisebach H. (1966). Biosynthesis of benzoic acids in *Gaultheria procumbens*. III. Z. Naturforsch. B.

[32] Ibrahim R.K. (1963). The distribution of o-pyrocatechuic acid in plants. Naturwissenschaften.

[33] Ibrahim R.K., Towers G.H.N. (1959). Conversion of salicylic acid to gentisic acid and o-pyrocatechuic acid, all labeled with carbon-14, in plants. Nature.

[34] Fujii K., Matsukawa T. (1936). Constitution of *Gaultheria miqueliana* Takeda. Yakugaku Zasshi.

[35] Kariyone T., Hashimoto Y. (1943). Microchemical detection of plant constituents. II. detection of arbutin. Yakugaku Zasshi.

[36] Yao G.M., Wang Y.B., Wang L.Q., Qin G.W. (2005). 3,5-Dihydroxy-2-hydroxymethyl-4*H*-pyran-4-one. Acta. Cryst..

[37] Eglinton G., Hamilton R.J., Martin-Smith M. (1962). The alkane constituents of some New Zealand plants and their possible taxonomic implications. Phytochemistry.

[38] Wang Y.F., Yao N., Tang B. (2005). Studies on antimicrobial activity of essential oil from the leaves of *Gaultheria yunnanensis*. Shanxi J. Tradit. Chin. Med..

[39] Li R.Q. (2005). A research abstracting methyl salicylate from the leaves of purpurea. J. Sichuan Vocat. Tech. Coll..

[40] Xiong Y.L., Xiao B., Ma X.J., Li C.L., Zheng J.H., Ye J. (2009). Effects of *Gaultheria yunnanensis* on adjuvant arthritis in rats. Chin. J. Chin. Mat. Med..

[41] Zhang M.F., Shen Y.Q. (2012). Research advances on hepatoprotective activities of oleanolic acid and ursolic acid. Anti. Infect. Pharm..

[42] Song C.W., Xiong L.D., Wang Y.J., Zhang N., Li X., Li Y., Li L., Yin S.F. (2012). Synthesis and inhibition of tyrosinase acitivity of new gentisic acid derivatives. Chin. J. Org. Chem..

[43] Chen S.Z. (1990). Analysis of chemical compositions of volatile oil from *Gaultheria yunnanensis* (Franch) Rehd. Chin. Tradit. Herb. Drugs.

[44] Kim J.H., Park Y.M., Shin J.S., Park S.J., Choi J.H., Jung H.J., Park H.J., Lee K.T. (2009). Fraxinellone Inhibits Lipopolysaccharide-Induced inducible nitric oxide synthase and cyclooxygenase-2 Expression by Negatively regulating nuclear factor-kappa B in RAW 264.7 macrophages cells. Biol. Pharm. Bull..

[45] Hiroaki O., Keiko Y., Keiji M., Tetsuo L., Munehiro N. (1997). Synthesis and biological activities of degraded limonoids, (±)-fraxinellonone and its related compounds. Tetrahedron. Lett..

[46] Wu Q., Ye C., Han W., Song P.L., Zou H.Y. (2007). Analysis of volatile oil in *Gaultheria yunnanensis* (Franch) Rehder. by SPME-GC-MS. J. Henan Univ. (Med. Sci.).

[47] Wang Y.F., Li X.F., Yu Z.X., Zhang Q.G., Yang X.H. (1992). The chemical constituents of the essential oil from the leaves of *Gaultheria leucocarpa* BL.var. *crenulata* (Kura.) T.Z. HSU. Acta Agric. Univ. Jiangxiensis.

[48] Ma X.Y., Wei T., Zhai J.J., Chen Y.Z. (1992). Analysis of chemical compositions of volatile oil from root of *Gaultheria yunnanensis* in Guiyang province. J. Instrumental. Anal..

[49] Yang J.S., Ma J.J., Yang L., Wu J.L. (1998). Studies on solid adsorption and stability of essential oil from pericarp of *Gaultheria yunnaensis* (Franch.) Rehd. Chin. J. Chin. Mat. Med..

[50] Tao C., Luo Y.N., Yang X.S., Wang D.P. (2011). Analysis of chemical compositions of volatile oil from root of *Gaultheria yunnanensis* (Franch) Rehd. J. Anhui. Agric. Sci..

[51] Zhang J.W., Guo X.Y. (1994). Analysis of chemical compositions of volatile oil of *Gaultheria yunnanensis* (Franch) Rehd. J. Guiyang Coll. Tradit. Chin. Med..

[52] Kim J., Seo S.M., Park  I.K. (2011). Nematicidal activity of plant essential oils and components from *Gaultheria fragrantissima* and *Zanthoxylum alatum* against the pine wood nematode, *Bursaphelenchus xylophilus*. Nematology.

[53] Adhikary S.R., Bashyal B.P. (1985). Aromatic plants of Nepal-part IV. essential oil from *Gaultheria fragrantissima* Wall. J. Nepal Pharm..

[54] Nikolić M., Marković T., Mojović M., Pejin B., Savić A., Perić T., Marković D., Stević T., Soković M. (2013). Chemical composition and biological activity of *Gaultheria procumbens* L. essential oil. Ind. Crop. Prod..

[55] Zhang B., He X.L., Ding Y., Du G.H. (2006). Gaultherin, a natural salicylate derivative from *Gaultheria yunnanensis*. towards a better non-steroidal anti-inflammatory drug. Eur. J. Pharmacol..

[56] Zhang D., Liu R., Sun L., Huang C., Wang C., Zhang D.M., Zhang T.T., Du G.H. (2011). Anti-inflammatory activity of methyl salicylate glycosides isolated from *Gaultheria yunnanensis* (Franch.) Rehder. Molecules.

[57] Zhang Z.Z., Guo D.A., Li C.L., Zheng J.H. (1999). Study on antibaeterial, anti-inflammatory and analgesic activities of *Gaultheria yunnanensis*. Northwest Pharm. J..

[58] Li D.C., Guo Z.Q., Lv H.N., She G.M. (2010). Antioxidant activity of extracts from *G.aultheria leucocarpa* var. *yunnanensis*. Acta Chin. Med. Pharm..

[59] Karuppusamy S., Muthuraja G., Rajasekaran K.M. (2011). Antioxidant activity of selected lesser known edible fruits from western ghats of India. Indian. J. Nat. Prod. Res..

[60] Ulyana M.A., Daniel E.A., Ma J., Michael H.N., Edward J.K. (2002). Antioxidant capacities of ten edible North American plants. Phytother. Res..

[61] Ma X.J., Zhao L., Du C.F. (2001). Screening of anti-bacteria activity of extracts of *Gaultheria leucocarpa* var. *yunnanensis*. Chin. J. Chin. Mat. Med..

[62] Middleton D.J. (1991). Infrageneric classification of the genus *Gaultheria* L. (Ericaceae). Botl. J. Linn. Soc.

[63] Hsu T.Z. (1981). Preliminary study of classification on Chinese Gaultheria. Acta. Bot. Yunnanica..

